# Hierarchically Structured Hybrid Membranes for Continuous Wastewater Treatment via the Integration of Adsorption and Membrane Ultrafiltration Mechanisms

**DOI:** 10.3390/polym15010156

**Published:** 2022-12-29

**Authors:** Roberto Scaffaro, Michele Gammino, Andrea Maio

**Affiliations:** Department of Engineering-Research Unit INSTM, University of Palermo, Viale delle Scienze, 90128 Palermo, Italy

**Keywords:** carbon nanotubes, graphene oxide, PVDF-HFP, hybrid nanocomposites, electrospinning, morphology, surface properties, mechanical properties, ultrafiltration, multifunctional

## Abstract

Growing environmental concerns are stimulating researchers to develop more and more efficient materials for environmental remediation. Among them, polymer-based hierarchical structures, attained by properly combining certain starting components and processing techniques, represent an emerging trend in materials science and technology. In this work, graphene oxide (GO) and/or carbon nanotubes (CNTs) were integrated at different loading levels into poly (vinyl fluoride-co-hexafluoropropylene) (PVDF-co-HFP) and then electrospun to construct mats capable of treating water that is contaminated by methylene blue (MB). The materials, fully characterized from a morphological, physicochemical, and mechanical point of view, were proved to serve as membranes for vacuum-assisted dead-end membrane processes, relying on the synergy of two mechanisms, namely, pore sieving and adsorption. In particular, the nanocomposites containing 2 wt % of GO and CNTs gave the best performance, showing high flux (800 L × m^−2^ h^−1^) and excellent rejection (99%) and flux recovery ratios (93.3%), along with antifouling properties (irreversible and reversible fouling below 6% and 25%, respectively), and reusability. These outstanding outcomes were ascribed to the particular microstructure employed, which endowed polymeric membranes with high roughness, wettability, and mechanical robustness, these capabilities being imparted by the peculiar self-assembled network of GO and CNTs.

## 1. Introduction

The polyvinylidene fluoride-co-hexafluoropropylene (PVDF-co-HFP) copolymer, owing to its excellent resistance toward thermal, photo–, and chemical degradation, as well as piezoelectric properties and hydrophobicity, is widely used for many functional applications, including cultural heritage conservation, wearable sensors, piezoelectric nanogenerators, biomedical devices, hydrogen production, and separation processes [1,2,3,4,5,6]. These latter applications are stimulating rising interest since environmental pollution and drinking water demand have become crucial global challenges [7]. Among the several water contaminants that are responsible for adverse effects on the environment and human health, aromatic dyes belong to the class known as emerging pollutants [8,9]. In fact, their removal from water is difficult due to their strong physicochemical stability in aqueous media, thus making most of the conventional water treatment techniques inefficacious [8]. Hence, advanced membrane separation strategies are gaining momentum. Among them, nanofiltration and ultrafiltration are the most encouraging possible strategies, both relying on the continuous operation of the process while differing from each other in terms of the particular mechanism of removal [8]. In fact, the former approach relies on size rejection, prompted by the presence of small pore diameters below hundreds of nanometers [8]. This feature, while maximizing removal efficiency, inevitably affects the membrane permeance, thus decreasing the flux of the permeate. The latter strategy, based on an electrostatic rejection mechanism, is preferable from the point of view of obtaining greater flow rates of water to be decontaminated [8]. In this context, materials science and technology researchers are urged to develop novel materials and/or processing technologies that are capable of achieving more and more efficient separation processes. For instance, the performance of PVDF-co-HFP membranes for the removal of molecular contaminants, such as dyes, is affected by pore size (usually being larger than those of the contaminant molecules), low flow rate, and surface fouling, due to their hydrophobic nature [10]. Hence, combining membrane filtration with adsorption is considered a promising approach to rapidly and efficiently treating large volumes of contaminated water [8]. In this case, active nanoparticles can be either embedded into the polymer matrix or even immobilized onto the membrane surface [8,11,12].

Indeed, integrating nanocarbon materials such as graphene oxide (GO) or carbon nanotubes (CNTs) into a PVDF-co-HFP matrix has been recently explored to further improve the filtering performance, by virtue of their interesting features [13,14,15]. In fact, beyond their well-known outstanding mechanical, electrical, and antioxidant properties [16,17], such nanoparticles possess a large surface area with plenty of potential active sites, thus being promising as nano adsorbents [18,19]. Another critical issue relates to the mechanical performance of ultrafiltration membranes. In fact, while high porosity favors rapid separation processes, conversely, the mechanical performance of materials tends to decrease [20]. In this context, the electrospinning technique gives rise to nanofibrous membranes with interconnected pores that are undoubtedly more porous than those achieved via wet phase inversion and other techniques but, on the other hand, their robustness is often unsatisfactory [20]. To solve this issue, mats prepared by electrospinning are usually stacked against each other and are thus welded by compression molding, with the ensuing increase of mechanical resistance at the expense of permeance [21].

A promising strategy to avoid the subsequent hot-pressing step could be the promotion and control of blobbing phenomena during electrospinning, by controlling the solvent-polymer phase separation rates. If the latter are slow enough to lead to the formation of an interconnected fiber network, the interlaminar adhesion of the mats could be improved, thus providing materials with considerable mechanical strength, despite their high porosity. Indeed, in a previous work [7], we demonstrated that the hybrid network formed by GO and CNTs may give rise to polymer nanocomposites with emergent features, including a strong affinity to methylene blue and methyl orange, and mechanical properties exceeding the sum of those of their constituent parts.

The aim of this work is to explore the feasibility of constructing highly porous electrospun mats that conjugate excellent performance in terms of permeance, pollutant rejection, and anti-fouling features, with outstanding mechanical robustness and durability. Hence, the structure-properties relationships of ternary nanocomposites, based on a PVDF-co-HFP matrix and a hybrid loading of GO and CNTs, were investigated and compared to those of binary systems containing solely GO or CNTs.

## 2. Materials and Methods

The GO and CNTs used in this work were synthesized in our laboratories, following the protocols reported in our previous studies [7,18,22,23,24,25]. Briefly, GO (lateral size < 45 μm, thickness = 0.7–0.8 nm, C/O ratio = 1.1, density = 1.76 g/cm^3^) was prepared by using Tour’s method, while CNTs (length = 1–2 μm, diameter = 10–20 nm, C/O ratio = 40.7, density = 2.24 g/cm^3^) were synthesized via fluidized bed chemical vapor deposition. The PVDF-co-HFP was a sample of Fluolyne HY, kindly supplied by CTS Europe (Altavilla Vicentina (VI), Italy). It is an elastomeric thermoplastic fluorinated copolymer with an Mw = 400,000 g/mol and a density equal to 1.77 g/cm^3^. Acetone and methylene blue (MB) of reagent grade were purchased from Sigma Aldrich (St. Louis, MO, USA).

The six formulations herein investigated are summarized in Table 1.

In order to obtain the designed formulation, nanoparticle(s) in the desired proportions were first dispersed in acetone via ultrasonication (4 h); thereafter, PVDF-co-HFP was poured (10 wt. %, with respect to the solvent) until homogeneous dispersion and then vigorously stirred until complete dissolution (6 h).

Each solution was then loaded into a glass syringe and electrospun using Linari equipment under the following operating parameters: flow rate, 1.8 mL/h, supplied high voltage, 15 kV, needle-to-collector distance, 12 cm, temperature, 25 °C, and relative humidity, 50%. Nanofibers were collected on an aluminum-coated rotary drum (diameter = 25 mm, speed = 25 rpm), for 180 min, in order to obtain ~50 μm–thick mats.

Morphology was investigated by combining different techniques. Scanning electron microscopy (SEM) was carried out by using an ESEM FEI QUANTA 200 microscope (Thermo Fisher Scientific, Waltham, MA, USA). Image analysis, performed by ImageJ 8 open-source software (https://imagej.nih.gov/ij/download.html, accessed on 25 November 2022) equipped with the Diameter J plug-in, was used to measure the size distribution of the fiber and pore diameter of the mats [26]. The overall porosity of the membranes was calculated via the gravimetric method, as typically used for PVDF-co-HFP electrospun mats [27]. Briefly, all pores of a membrane sample of known weight and dimensions were completely filled with ethanol by soaking the mats for 2 h. Porosity was then calculated according to Equation (1):

(1) $$\mathrm{Porosity}\;\left(\%\right)=\frac{W_{s,\mathrm{wet}}-W_{s,\mathrm{dry}}}{\rho_{L}V_{s}}\times 100$$

where $W_{s,\mathrm{wet}}$ and $W_{s,\mathrm{dry}}$, respectively, indicate the weight of the wet and dry membrane samples, $\rho_{L}$ is the density of the wetting liquid, and $V_{s}$ is the sample volume.

Atomic force microscopy (AFM) was employed to measure the roughness of the membranes. AFM measurements were performed in air using a Bruker FAST–SCAN microscope equipped with a closed-loop scanner in the soft tapping mode, using a probe with an apical radius of about 5 nm. Arithmetic average roughness (Ra) and quadratic mean roughness (Rq) were calculated on the squared areas (side length = 1 µm).

Sessile drop water contact angle (WCA) testing was carried out to assess the surface wettability of membranes. Experiments were performed at room temperature, using an FTA 1000 (First Ten Ångstroms, Cambridge, UK) instrument. First, 4 μL of deionized water was dropped onto the surface of each sample by way of an automatic liquid drop dosing system. Images of the drops onto the surface were acquired after 20 s. Mechanical testing was performed using an Instron 3365 dynamometer (Instron, Norwood, MA, USA) on the prismatic specimens with a width = 10 mm, length = 90 mm, and thickness ~50 µm (measured before each measurement). The tests were performed on at least 10 replicates, under the following conditions: distance between the jaws = 30 mm, crosshead speed = 1 mm min^−1^ until failure [28]. The salient data were provided as mean values ± standard deviations. Elastic modulus (E_1_) was calculated as the slope of the stress–strain curve in the initial linear range (E_1_). The slope of each curve was also calculated in the strain-hardening region (E_2_). Toughness was measured as the integrated area of each curve. Tensile strength (TS) and elongation at break (EB%) were calculated, respectively, as the ordinate and the abscissa of the stress-strain curve at failure.

### Fabrication and Characterization of Devices for Ultrafiltration

**Figure 1:**
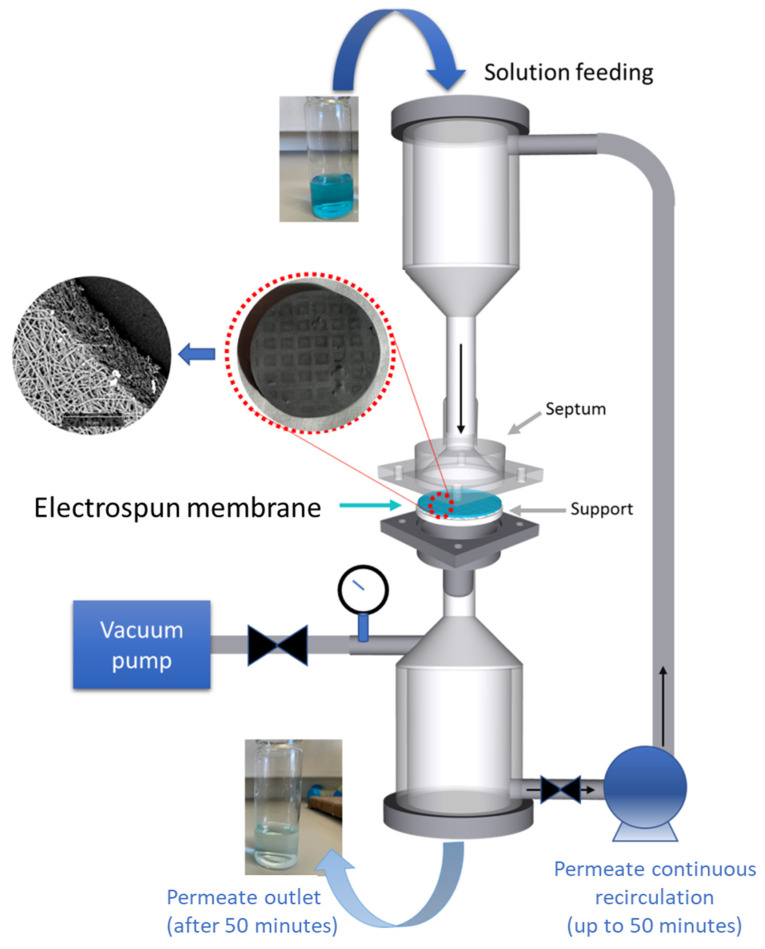
Schematics of the experimental setup and the device used, along with digital and SEM photographs of a membrane.

Devices for ultrafiltration were assembled as depicted in Figure 1. The membrane septum and support grid were homemade and were fabricated by rapid prototyping. Thereafter, the support grid was placed onto electrospun membranes (still adherent to aluminum foil) and ethanol was poured in to allow the easy detachment of the mats from the aluminum foil and their subsequent placement on the grid.

A dead-end configuration was designed, with the solution being forced to flow across the membrane by vacuum pumping. Each treatment cycle involved the numerous and rapid passage of the permeate through the membrane, ensured by continuous recirculation for 50 min. Thereafter, the permeate was withdrawn, while the membrane was regenerated in methanol and then reused for the next cycle.

Water permeance of the membranes was assessed at room temperature by performing flux experiments in the aforementioned home-made setup. Membranes of 45 mm in diameter were fixed into a test cell, then a dead-end vacuum filtration system was used at an absolute pressure of 85 kPa, and pure water flux (J; L/m^2^h) was calculated using Equation (2):

(2) $$J=\frac{V}{A\;t\;}$$

where V is the permeate volume, A is the effective cross-sectional area of the membrane, and t is the elution time (h).

During such tests with deionized water, it was also possible to determine the membrane pore diameters in the bulk, according to Equation (3):

(3) $$r_{m}=\sqrt{\frac{\left(2.9-1.75\varepsilon \right)\times 8\mu \mathrm{lQ}}{\varepsilon \times \mathrm{TMP}\times A}}$$

where μ is the viscosity of permeated water (8.9 × 10^−4^ Pa s), Q is the flux of permeated water through the membrane per unit time (m^3^ s^−1^), TMP is the transmembrane pressure (Pa), and l and A are, respectively, the thickness and the effective area of the membrane.

In order to quantify MB removal, vacuum filtration tests under the same conditions were carried out on 100 mL aqueous MB solutions (5 mg/L). MB concentration in the feeding solution and in the filtrate was then measured via UV-vis spectroscopy (at λ = 667 nm) and the ultimate separation efficiency, R (%), was calculated according to Equation (4):

(4) $$R\;\left(\%\right)=\left(1-\frac{A_{2}}{A_{1}}\right)\times 100$$

where A_2_ and A_1_ are the values of absorbance of methylene blue, recorded at the end and at the beginning of the vacuum-filtration experiments, respectively.

As stated above, each membrane was subjected to vacuum-filtration runs with the continuous recirculation of permeate up to 50 min before washing, and the removal efficiency of each sample was also measured at predetermined time intervals, in order to investigate its time-dependent behavior during each cycle. The analysis of anti-fouling properties was conducted by calculating the water flux recovery ratio (FRR), along with reversible ($R_{r}),$ irreversible ($R_{ir})$ and total ($R_{t})$ fouling rations, which are established using Equations (5)–(8):

(5) $$\mathrm{FRR}\;\left(\%\right)=\frac{J_{w2}}{J_{w1}}\times 100$$

(6) $$R_{r}\;\left(\%\right)=\frac{J_{w2}-J_{p}}{J_{w1}}\times 100$$

(7) $$R_{ir}\;\left(\%\right)=\frac{J_{w1}-J_{w2}}{J_{w1}}\times 100$$

(8) $$R_{t}\;\left(\%\right)=\frac{J_{w1}-J_{p}}{J_{w1}}\times 100$$

where $J_{w1}$ is the flux of pure water passing through the membrane, $J_{p}\;$ is the flux of the water containing the pollutant, and $J_{w2}\;$ is the flux of pure water passing through the membrane after it has been cleaned. Reusability was evaluated by subjecting the membranes to 10 process cycles. At the end of each cycle, lasting 50 min, the membranes were regenerated by washing with methanol, and eventual changes in separation efficiency or mechanical damage were recorded.

## 3. Results and Discussion

### 3.1. Morphology and Surface Features of the Electrospun Mats

**Figure 2:**
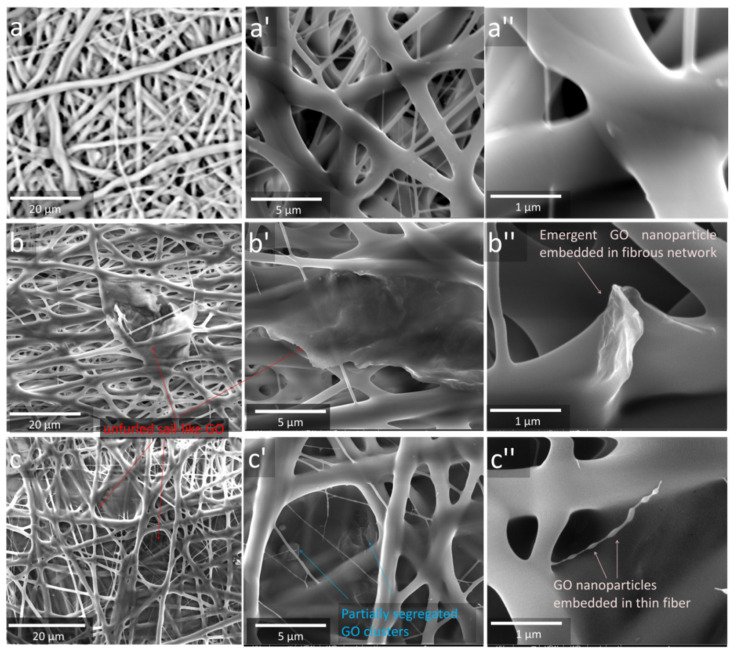
SEM micrographs at different magnifications of C0–G0 (**a**–**a”**), C0–G1 (**b**–**b”**), C0–G2 (**c**–**c”**). Arrows indicate the peculiar morphologies imparted by nanoparticles.

**Figure 3:**
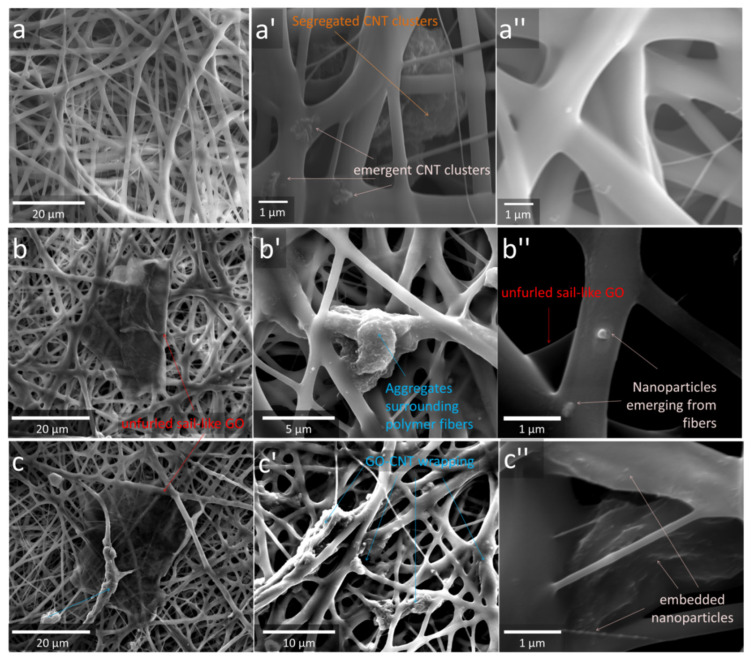
SEM micrographs at different magnifications of C2–G0 (**a**–**a”**), C2–G1 (**b**–**b”**), and C2–G2 (**c**–**c”**). Arrows indicate the peculiar morphologies imparted by nanoparticles.

The morphology of the mats was investigated via SEM and image analysis. Figure 2 shows the SEM micrographs at different magnifications of C0–G0 (a–a”), C0–G1 (b–b”), and C0–G2 (c–c”), whereas Figure 3 provides those of C2–G0 (a–a”), C2–G1 (b–b”), and C2–G2 (c–c”). Table 2 provides the salient features of fibrous architectures, that is, the mean fiber and pore diameter of the top surface, calculated via image analysis (for size distributions, see Figure S1 in the Supplementary Materials) and membrane porosity, evaluated by the gravimetric method.

As one can see, all the mats, i.e., the neat polymer and its nanocomposites, show either a weaker or more pronounced blobbing phenomena, which led to the formation of an interconnected fibrous network. This occurrence can be ascribed to the use of acetone only to prepare polymeric solutions, which resulted in slow phase separation rates. Neat polymer (Figure 2a–a”) exhibits fibers with a smooth surface and a discrete diameter distribution, with 45% of fibers displaying a submicrometric size, 43% having a diameter of between 1.6 and 3.2 µm, and 12% being thicker than 4 µm (Figure S1 in the Supplementary Materials). This aspect can be ascribed to the high charge density of PVDF-co-HFP, which is known to promote the splitting of the jet, thus forming both thicker and thinner fibers [29]. GO addition, while not significantly altering the fiber diameter, was found to promote the formation of blobs to a greater extent, with the consequent increase in fiber junctions. This is most likely caused by its well-known tendency to hinder solvent evaporation [30]. Moreover, filler dispersion proved to be inhomogeneous, with clearly visible aggregation events (see Figure 2b–c). However, it is worth noting that GO foils display a broad distribution of lateral sizes, ranging from hundreds of nanometers to tens of micrometers. Larger GO foils were found to protrude out of the fibrous network, with an unfurled sail-like configuration. A closer inspection of these latter forms in the case of C0–G1 and C0–G2 is provided in the detailed micrographs of Figure 2b’–c’, respectively, which show GO sheets that are eventually surrounded by the polymer.

The submicrometric sheets were small enough to be integrated within the fibrous cage (Figure 2b”–c”) and were found to be either emergent from the fiber surface or embedded inside the fibers. Hence, both samples show an extremely variegated microstructure, comprising a cage made of fibers of different diameters, with GO particles of different lateral sizes, which, in turn, can be found either outside or integrated within the fibrous structure.

The morphology of the system C2–G0 is shown in Figure 3a–a”. In this case, the addition of CNTs alone did not significantly alter the fibrous architecture of the neat polymer, which retained its random orientation and discrete, multimodal size distribution, with substantially similar values of average fiber diameter, although the filler dispersion proved to be inhomogeneous, with easily detectable nanoparticle clusters, likely arising from their scarce dispersibility in acetone. It is worth noting that such aggregates were localized in both the intra- and inter-fiber regions (see Figure 3a’). As visible from the micrographs of C2–G1 (Figure 3b–b”) and C2–G2 (Figure 3c–c”), the hybrid loading of CNTs and GO determined some changes in the mat morphology. Differently from the systems containing solely GO or CNTs, a drastic reduction in the size and number of aggregation events was observed in the hybrid electrospun mats. The fiber diameter distribution of C2–G1 and C2–G2 (Figure S1 in the Supplementary Materials) proved to be narrower than those of the other systems, and the fibers displayed the lowest values of the mean diameter (Table 2). The presence of CNTs also affected the configuration of the larger GO sheets, which were found to either stretch out like unfurled sails (Figure 3b–c) or even to roll up around their axes, thus wrapping the polymeric fibers (Figure 3b’–c’). These two phenomena were observed in both the hybrid samples, although both showed a dose-dependent trend: the former proved to prevail at low GO loading (C2–G1, Figure 3b–b”), while the latter governed the morphology of the samples containing the highest GO content (C2–G2, Figure 3c–c”). It should also be noted that the hybrid samples showed a higher concentration of nanoparticles emerging from the fiber surface and that the unfurled sail-like GO lamellae displayed a thinner thickness (Figure 3c–c”), likely because the presence of CNTs disturbed the typical stacking of the GO layers.

**Figure 4:**
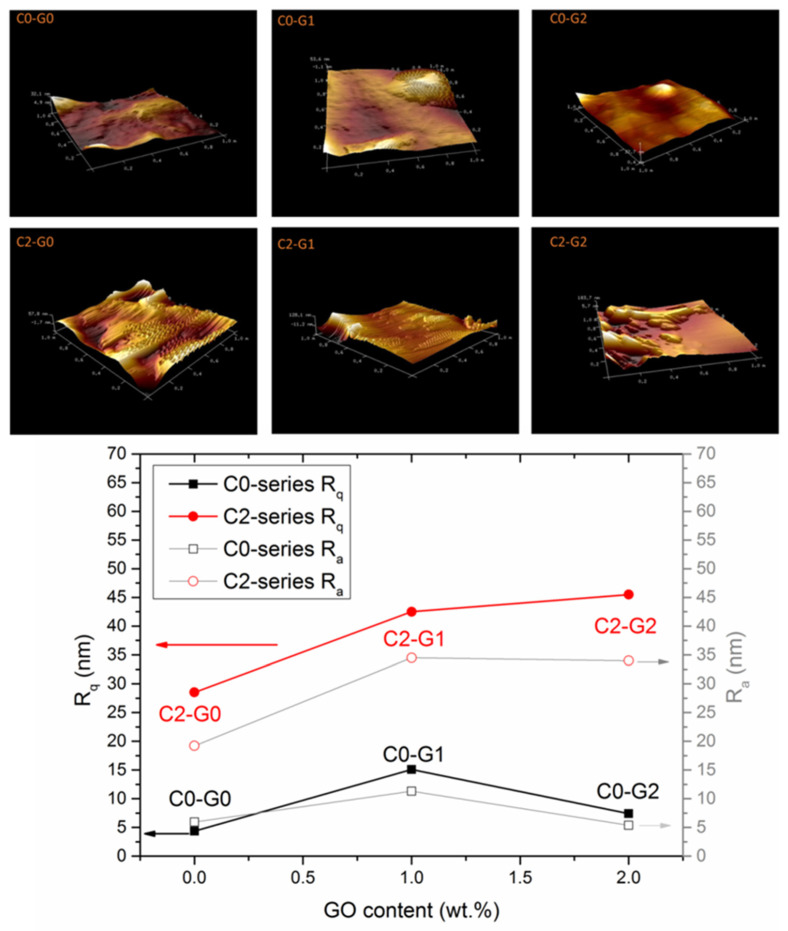
AFM images of the samples, together with the quadratic (left Y–axis) and arithmetic (right Y–axis) mean roughness, plotted as a function of GO content for both the C0–series and C2–series nanocomposites.

In order to derive more information about the surface morphology of the fibers, AFM analysis was carried out and the images were recorded on squared regions of 5 µm and 1 µm per side, as reported in Figure S2 in the Supplementary Materials and Figure 4, respectively, along with the values of the arithmetic average and squared mean roughness of the samples, reported in the bottom panel of Figure 4.

It can be seen that the presence of nanocarbons alters the surface features of the fibers, although this is seen to different extents, depending on the formulation. As already envisaged from the SEM analysis, in fact, nanoparticles could be found either emerging from fibers or covered with polymeric layers. In C0–G1, it is easy to recognize the typical rough texture imparted by two distinct GO foils, emerging from the surface of a smooth fiber. In C0–G2, the fiber surface appears to be as smooth as that of neat polymer, likely because most of the foils proved to be surrounded by a thick polymeric layer, in agreement with the results of the SEM imaging. In the case of C2–G0, instead, AFM was able to detect the presence of CNTs emerging from the fiber surface, which resulted in a brush-like morphology. The C2–G1 and C2–G2 samples displayed an extremely rough texture, likely due to the formation of an extensive GO-CNT hybrid framework that might have promoted a higher dispersion degree and, thus, the localization of a higher concentration of nanoparticles in the surface of fibers. In fact, as is visible in Figure 4, all membranes containing GO possess a fiber roughness similar to or moderately greater than that of pristine PVDF-co-HFP, likely due to bad filler dispersion, which results in smooth polymeric fibers and discrete aggregates. On the other hand, the C2–series samples display a rougher texture, with the C2–G2 sample showing the largest Rq and Ra values, from almost equal to double those of C2–G0, and up to 700% higher than those of neat C0–G0.

**Figure 5:**
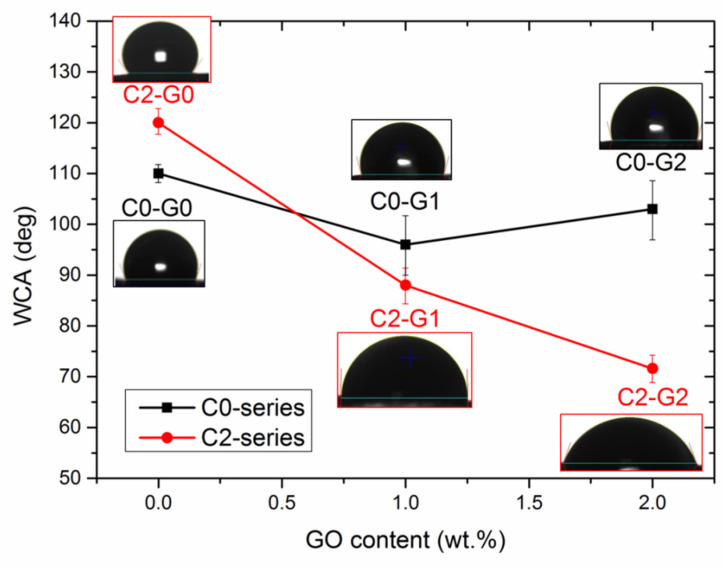
The hydrophilicity of the samples, as evaluated by WCA testing.

Surface wettability was studied via WCA testing and the results are provided in Figure 5.

In the C0–series systems, the hydrophobic character of PVDF-co-HFP (WCA = 108°) is substantially maintained, even in the presence of GO, despite the well-known hydrophilicity of the latter. Again, this result could be likely ascribed to the poor extent of filler dispersion, as is also verified by the high data scattering of C0–G1 and C0–G2, and to the scarce presence of nanoparticles emerging from the surface, in full agreement with the SEM and AFM observations. Predictably, the hydrophobic character of the polymer was further enhanced by adding CNTs, due to their hydrophobicity, even being boosted by the brush-like morphology of the fibers. However, incorporating GO together with CNTs led to different behavior. WCAs were found to decrease as a function of the GO content, until the hydrophilic character of the latter prevailed over the hydrophobic one of the other components at the highest GO dose (C2–G2), thus resulting in a WCA as low as 71°. These results are in strong agreement with those of the SEM and AFM measurements (see, again, Figure 2, Figure 3 and Figure 4).

### 3.2. Mechanical Properties of Electrospun Mats

**Figure 6:**
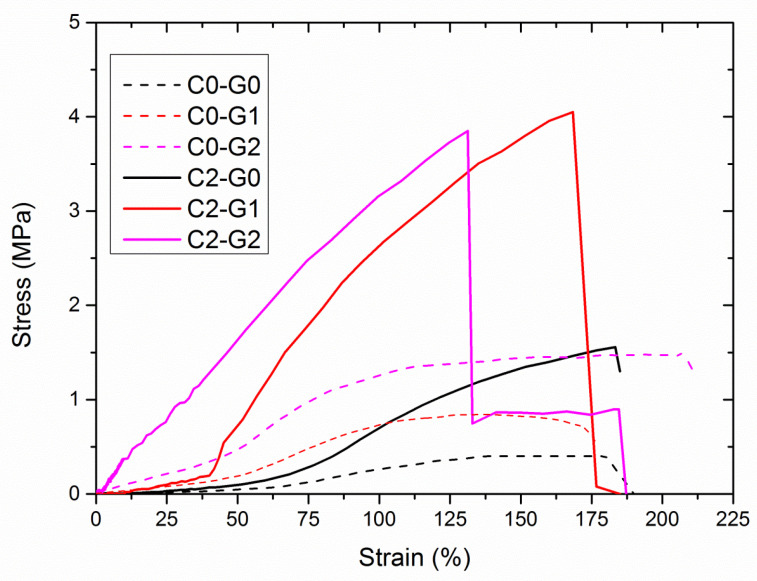
Representative stress–strain curves of the membranes during tensile testing.

The representative stress–strain curves collected during tensile tests are provided in Figure 6, whereas the salient mechanical properties measured via tensile tests are summarized in Table 3. It can be noted that neat polymer and its nanocomposites, with the only exception of the C2–G2 sample, display a J-shaped strain-stiffening behavior, being soft and compliant at small strains while becoming rapidly stiffer at higher strains. In fact, as is visible in Table 3, such samples show an initial slope of the stress–strain curve (E_1_), which is quite significantly lower than that calculated in the strain-hardening region (E_2_). Indeed, this peculiar mechanical behavior, likely attributable to the multimodal distribution of fiber diameters (Figure S1 in the Supplementary Materials), was already detected for other fibrous systems, based on PVDF and PVDF-co-HFP [31].

The mechanical behavior of C0–G1 and C0–G2 proved to be dominated by the matrix, although the presence of nanofillers imparted strengthening and stiffening effects, without compromising ductility. C2–G0 shows an ultimate resistance and deformability similar to those of C0–G2, but the J-shape of the curve is more pronounced, likely because of the orientation of CNTs along the deformation axis, with the resulting strain-hardening. In C2–G1, which displayed the highest TS, the formation of a GO-CNT hybrid network and the prevalent unfurled sail-like configuration of GO reasonably resulted in a remarkable aliquot of GO-CNT hybrids, oriented along the deformation axis, with consequent strain-hardening that led to the most remarkable J-shaped behavior. In C2–G2, instead, the mechanical behavior seemed to be governed by the GO-CNT hybrid framework, which endowed the fibers with higher stiffness even at low strains, thus suppressing the J–shaped strain-stiffening behavior. The addition of GO and/or CNTs, therefore, determined the significant stiffening and strengthening of the PVDF-co-HFP matrix, without compromising its ductility. In particular, hybrid membranes retain the good deformability seen in neat PVDF-co-HFP (with an EB close to 200%), while experiencing an outstanding increase in tensile strength (20 times higher than that of neat polymer and 2–3 times higher than those of the nanocomposites containing solely GO or CNTs), and toughness (almost 9 times higher than that of PVDF-co-HPF and about double or triple those of the composites containing only one type of nanofiller), thus possessing mechanical prerequisites that are good enough to be used for ultrafiltration. Indeed, the values of the tensile strength of these materials match those of less porous membranes, prepared via wet phase inversion or compression molding-aided assembly [32,33,34].

### 3.3. Performance of Electrospun Membranes toward Water Decontamination

We then assessed the possibility of using such membranes for decontaminating water from MB, chosen as the proof of the concept. In fact, it is well known that CNTs and, especially, GO have a strong affinity to cationic dyes such as MB, owing to the electrostatic interactions between the negatively charged surface of the nanocarbons and the positive dipole (=N^+^H group) from the dye molecules, along with aromatic interactions via π–π stacking [35,36]. Water flux and MB retention experiments yielded useful information about the permeability and selectivity of the membranes. Unfortunately, because of their poor mechanical performance, C0–G0 and C0–G1 experienced mechanical failure during the preliminary flux tests in pure water, whereas C0–G2 underwent perforation after 3 min of filtration experiments with water contaminated by MB dye. Therefore, the characterization was restricted to C2–G0, C2–G1, and C2–G2.

**Figure 7:**
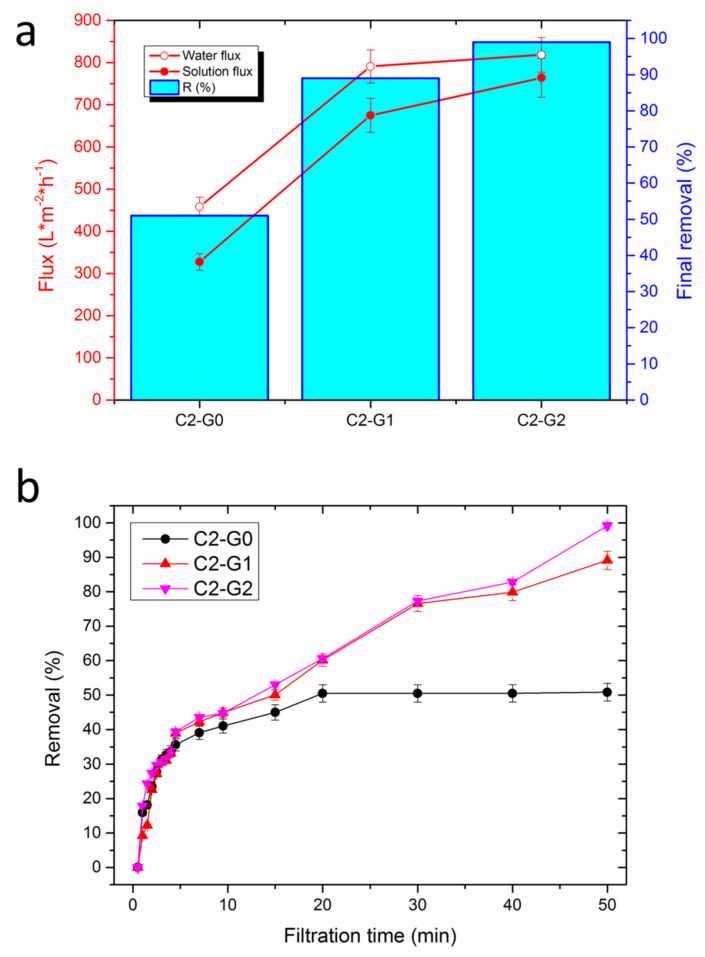
(**a**) Water and solution flux, along with the final removal of MB, as a function of the GO content of hybrid membranes; (**b**) removal as a function of filtration time, for the three systems investigated.

Figure 7a provides the permeance of membranes toward pure water (empty circles), and solution flux (filled circles), along with the final removal rate of MB dye (histograms). The outcomes indicate that all these samples display good water permeance, which was proven to remain stable during the experiment, due to the high porosity levels of the electrospun fibrous mats, along with the suitable pore size. In fact, the progressive deposition of electrospun fibers onto each other led to a distinctive pore architecture, consisting of micrometric pores on the external surface, as calculated via image analysis (see, again, Table 1, row no. 2), and with smaller pores in the membrane bulk, with diameters in the order of tens of nanometers, as measured according to Equation (3) (see row no. 4 of Table 2). Nevertheless, the mean pore diameters (ca. 30–40 nm) of these membranes are still significantly larger than the Stokes diameter of MB molecules (1–2 nm).

**Figure 8:**
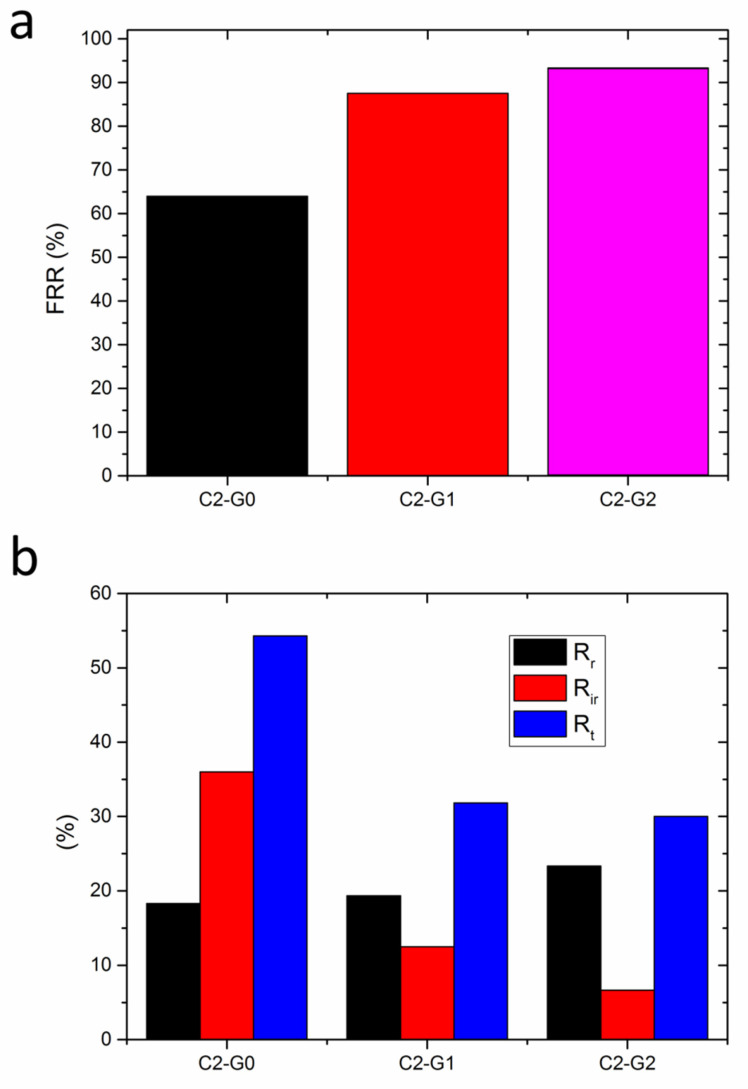
Anti-fouling performance of the membranes: (**a**) flux recovery ratio (FRR); (**b**) the ratios of reversible (R_r_), irreversible (R_ir_), and total (R_t_) fouling.

However, a clear trend was observed as a function of GO content, reasonably ascribed to the different surface characteristics of each sample, including the wettability and roughness. It is noteworthy that C2–G2 yielded the highest flux of pure water (as large as 820 L × m^−2^ h^−1^), which was far superior to the vacuum-ultrafiltration membranes recently proposed for treating water contaminated by MB dye [37,38]. C2–G1 showed slightly lower permeance (780 L × m^−2^ h^−1^), whereas C2–G0 offered the worst performance (450 L × m^−2^ h^−1^). This aspect put into evidence the crucial role of oxygen-rich GO lamellae, which boost the permeability of the membranes, owing to their hydrophilicity (Figure 5) and the wrinkled texture of GO foils, with the consequent enhancement of roughness (Figure 4). When subjected to the flux of water contaminated by MB dyes, the differences between C2–G1 (660 L × m^−2^ h^−1^) and C2–G2 (770 L × m^−2^ h^−1^) tended to augment. Nevertheless, both samples displayed J_p_ values that were quite higher than those of C2–G0 (320 L × m^−2^ h^−1^). Thus, incorporating GO and CNTs into a PVDF-co-HFP electrospun membrane results in extremely robust materials that can withstand water flux without failure, despite their high porosity and thin fibers. This latter aspect allows outstanding and constant permeability, which was granted by the combination of hydrophilicity (Figure 5) and by adequate pore size (see Figure S3 in the Supplementary Materials and Table 2). Often, increasing permeability can lead to a worsening of the dye rejection performance. In this case, instead, C2–G2 also showed the highest capacity to reject MB cationic dyes (99%), followed by C2–G1 (89%), while C2–G0 (51%) displayed the worst results. Furthermore, the analysis of the evolution of removal efficiency upon treatment time (Figure 7b) indicates that the C2–G0 membranes achieved a plateau within 20 min, thus suggesting the possible insurgence of fouling issues. These latter problems, while not affecting the flux that is mainly governed by larger pores, are detrimental to the adsorptive removal of dyes, since the binding sites tend to be saturated. In contrast, the removal efficiency of hybrid membranes proved to monotonically increase over time, because of the larger extent of the active surface available for MB adsorption. Indeed, the main drawbacks of dead-end filtration generally include recurring production interruptions and product losses due to backwashing or even the replacement of the filter elements, caused by either pore occlusion, which would determine the flow decay, or the saturation of active sites, with the consequent loss of removal performance after a certain time. Hence, one of the most critical design parameters is the fouling resistance of the membranes to water treatment. This aspect was assessed, and the results are shown in Figure 8, which displays the flux recovery ratio (FRR) (Figure 8a), together with the irreversible, reversible, and total fouling ratios (Figure 8b).

The trend observed for FRR follows that seen for WCA, with the most hydrophilic C2–G2 membranes displaying the strongest anti-fouling properties, i.e., the highest flux recovery ratio (93.33%), followed by C2–G1 (88%), whereas C2–G0 demonstrated the worst performance (64%).

It is noteworthy that the aliquot of irreversible fouling is strikingly low for C2–G2 (6%), which displays promising reusability. In contrast, the mats containing CNTs only (C2–G0) are prone to irreversible fouling, which is likely due to their hydrophobicity and to the strong tendency of CNTs to form stable complexes with MB molecules via π–π stacking [7], along with the progressive occlusion of pores.

**Figure 9:**
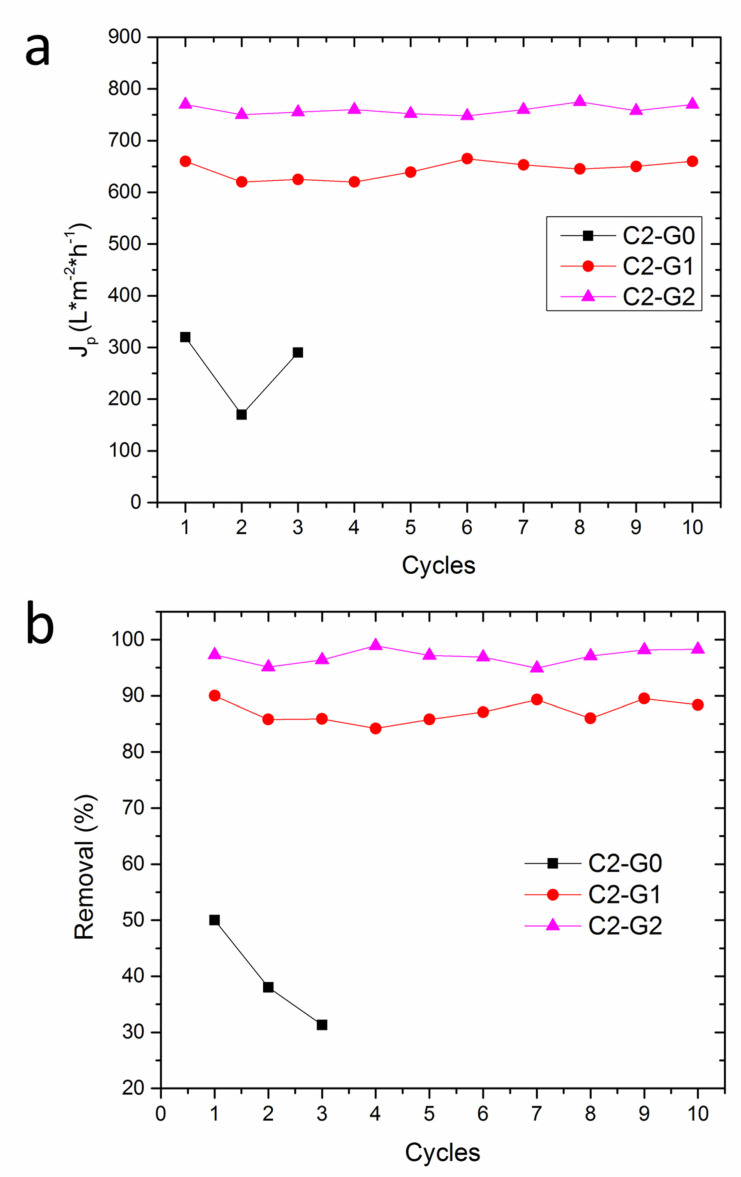
Reusability of the membranes in terms of flux (**a**) and removal (**b**), for up to 10 cycles.

The reusability of the systems is another crucial prerequisite in the perspective of reducing economic and environmental costs. The results of these tests, shown in Figure 9, provide evidence that C2–G0 experienced damage after 3 cycles, whereas C2–G1 and C2–G2 were proven to withstand mechanical stresses without failure over the whole time period investigated.

These outcomes, which are in strong agreement with those of the mechanical testing, once again point out, on the one hand, the difficulty of achieving efficient membranes via electrospinning and, on the other hand, the surprising reinforcing effect exerted by the hybrid GO-CNT network integrated within the polymer matrix. Beyond the considerations of mechanical durability, it is worth noting that hybrid membranes substantially retained a constant flux and a high separation efficiency, even after 10 cycles, while the C2–G0 sample has undergone a dramatic decline in separation performance, most probably because of its poor anti-fouling properties. In fact, in this latter sample, an initial flux decrease was observed during the second cycle, likely because of fouling and the partial occlusion of pores. These issues can be explained, considering that C2–G0 displays the strongest hydrophobicity and the smallest mean pore size in the bulk (see again the last row of Table 2). On the other hand, the raising of the flux detected in the third cycle, associated with a further loss of separation efficiency, presumably indicates the incipient structural failure of the mats.

It is worth noting that the pore architecture substantially remained stable in all the cases (the SEM results are not reported for the sake of conciseness), because the fibers are physically interconnected by junctions. Nevertheless, in response to the severe conditions, the less mechanically robust membranes underwent macroscopic perforation, while the hybrid membranes remained unaltered.

**Figure 10:**
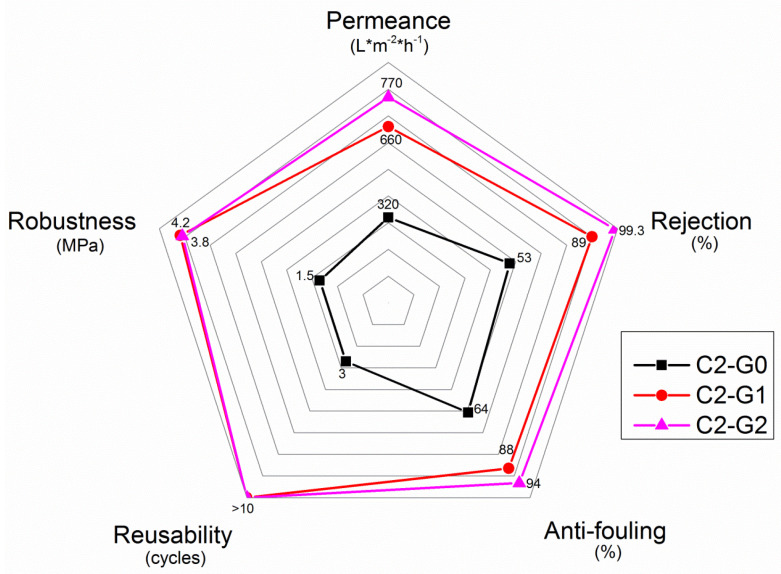
Radar-plot analysis of the overall performance of the membranes investigated.

Ultimately, a synoptic view of the overall performance of the three systems is provided as a radar plot in Figure 10.

As one can see, hybrid membranes (especially C2–G2) displayed the best performance, in terms of mechanical robustness, permeance, removal capacity, anti-fouling properties, and reusability, showing values at least double those of the mats containing CNTs only.

## 4. Conclusions

Electrospun membranes, based on PVDF-HFP loaded with GO and/or CNTs, were constructed by avoiding toxic solvents and surfactants, exploiting the slow-phase separation of polymers in acetone to promote blob formation. Generally, electrospun mats are produced by preparing polymer solutions in a mixture containing a solvent and a non-solvent. The latter help to increase the rapidity of phase separation, which leads to the formation of single fibers. In this case, instead, we decided to promote blobbing in order to construct a grid of interconnected fibers, to provide thin membranes with mechanical strength high enough to withstand the mechanical stresses caused by solution flow and the vacuum. It is worth noting that generally, the proposed electrospun membranes are prepared in at least two steps: (i) membrane fabrication via electrospinning, using a solvent/non-solvent couple; (ii) further consolidation of the mats via hot-pressing, to merge the fibers with each other so as to improve the mechanical performance.

In this case, our method is faster and easier, since we exploit the slow phase separation of the PVDF-HFP/acetone system, with the residual solvent promoting the one-step formation of interconnected fibrous grids.

Morphological analysis established that the hybrid loading of GO-CNTs led to uniform dispersion, showing the formation of a well-structured nanocarbon framework throughout the matrix, with several nanoparticles emerging from the fiber surface. By virtue of this feature, hybrid membranes displayed the highest values of roughness and hydrophilicity, beyond that of outstanding mechanical robustness, coupled with high porosity and micrometric pore diameter. Hence, when tested as membranes for vacuum-assisted ultrafiltration, they showed the highest values of permeance (800 L × m^−2^ h^−1^) and the capability of removing methylene blue (99%), with negligible fouling issues (irreversible and reversible fouling below 6% and 25%, respectively) and remarkable reusability of up to at least 10 cycles.

## Figures and Tables

**Table 1:** Formulation of the samples investigated (amounts given in wt %).

Sample Codename	C0–G0	C0–G1	C0–G2	C2–G0	C2–G1	C2–G2
PVDF-co-HFP	100	99	98	98	97	96
CNTs	0	0	0	2	2	2
GO	0	1	2	0	1	2

**Table 2:** Characterization of the fibrous architecture of the mats.

Property	C0–G0	C0–G1	C0–G2	C2–G0	C2–G1	C2–G2
Mean fiber diameter ^1^ (µm)	1.8	1.9	1.82	1.6	0.6	0.8
Mean pore diameter of the external surface ^1^ (µm)	2.38	0.44	0.3	2.51	1.73	2.74
Porosity ^2^ (%)	87.8	81.2	82.4	90.1	89	91.6
Mean pore diameter in the bulk ^3^ (nm)	N/A	N/A	N/A	28–30	34–37	37–40

^1^ Measured by image analysis on the top surface; ^2^ assessed by gravimetric analysis, using Equation (1); ^3^ calculated by flow experiments with pure distilled water, according to Equation (3).

**Table 3:** Salient tensile properties of the samples.

Property	C0–G0	C0–G1	C0–G2	C2–G0	C2–G1	C2–G2
E_1_ (MPa)	0.104 ± 0.00	0.323 ± 0.01	0.824 ± 0.01	0.197 ± 0.01	0.485 ± 0.03	4.0 ± 0.2
E_2_ (MPa)	0.43 ± 0.01	0.95 ± 0.07	2.4 ± 0.06	1.8 ± 0.02	7.2 ± 0.2	3.81 ± 0.03
TS (MPa)	0.41 ± 0.01	0.84 ± 0.01	1.48 ± 0.03	1.56 ± 0.04	4.05 ± 0.1	3.85 ± 0.16
EB (%)	178 ± 18	175 ± 11	215 ± 10	190 ± 15	180 ± 19	183 ± 22
Toughness (MJ/m^3^)	0.4 ± 0.01	0.88 ± 0.01	2.21 ± 0.03	1.21 ± 0.02	3.47 ± 0.06	3.22 ± 0.07

## Data Availability

The data presented in this study are available on request from the corresponding authors.

## Supplementary Materials

The following supporting information can be downloaded at: https://www.mdpi.com/article/10.3390/polym15010156/s1, Figure S1: Size distribution of fiber diameter for the samples investigated; Figure S2: Three-dimensional view of the AFM images, recorded on squared regions (5 µm per side) of the samples; Figure S3: Pore diameter distribution of the membranes, measured by image analysis on the top surface.
