# Supplementary material for: Hierarchically Structured Hybrid Membranes for Continuous Wastewater Treatment via the Integration of Adsorption and Membrane Ultrafiltration Mechanisms
Source: Polymers (Basel). 2022 Dec 29;15(1):156. doi: 10.3390/polym15010156 (PMC9824439; doi:10.3390/polym15010156)
Supplement: Supplementary file 1 [file polymers-15-00156-s001.zip › polymers-2094922-supplementary.pdf]

polymers

MDPI

Article

Hierarchically Structured Hybrid Membranes for Continuous Wastewater Treatment via the Integration of Adsorption and Membrane Ultrafiltration Mechanisms

Roberto Scaffaro [1,*], Michele Gammino [1] and Andrea Maio [1,*]

[1] Department of Engineering-Research Unit INSTM, University of Palermo, Viale delle Scienze, 90128 Palermo, Italy

* Correspondence: roberto.scaffaro@unipa.it (R.S.); andrea.maio@unipa.it (A.M.)

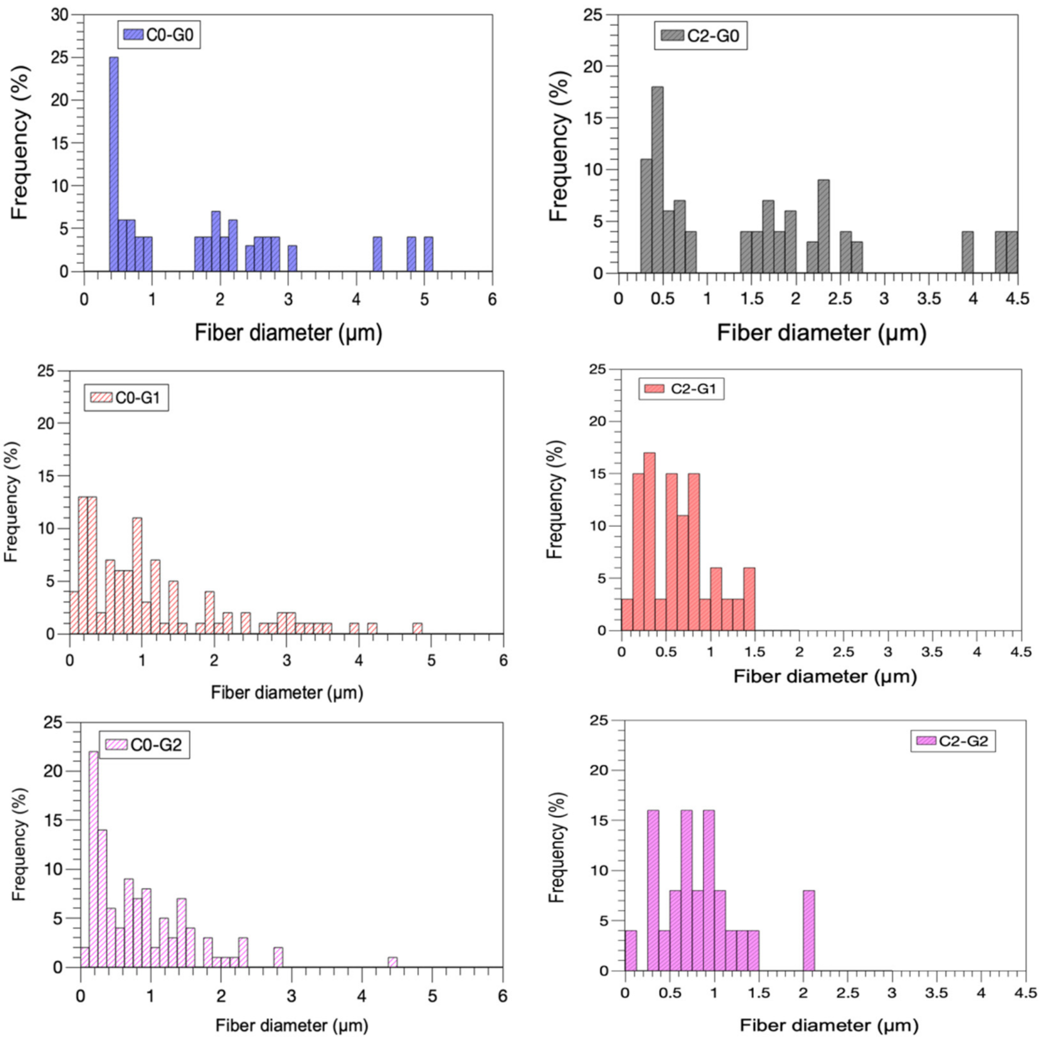

Figure S1. Size distribution of fiber diameter for the samples investigated.

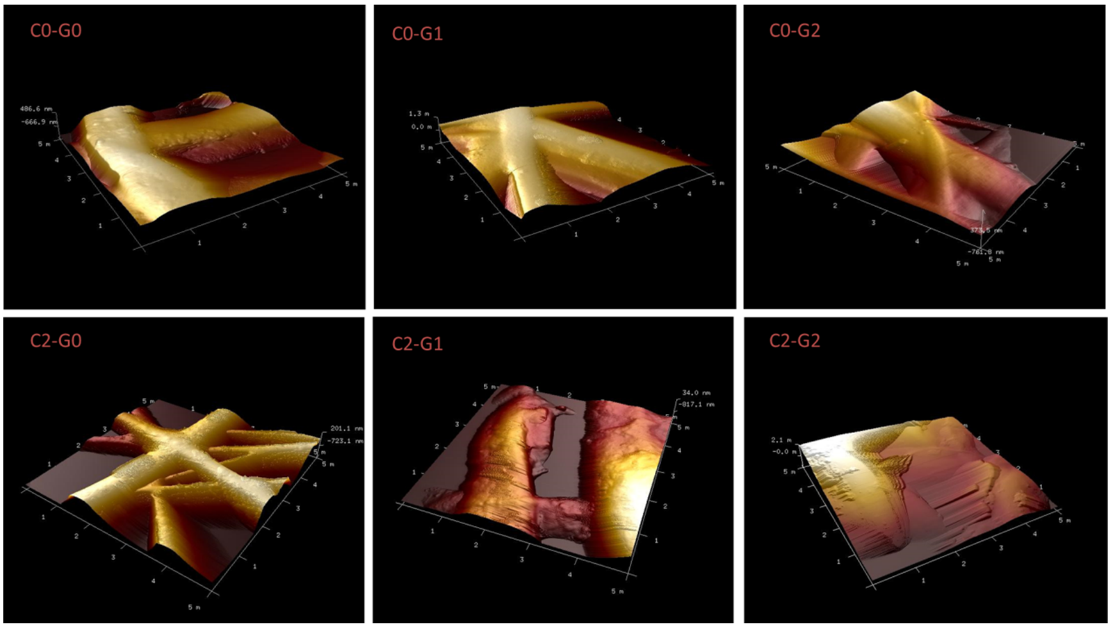

Figure S2. 3D view of AFM images recorded on squared regions (5 μm per side) of the samples.

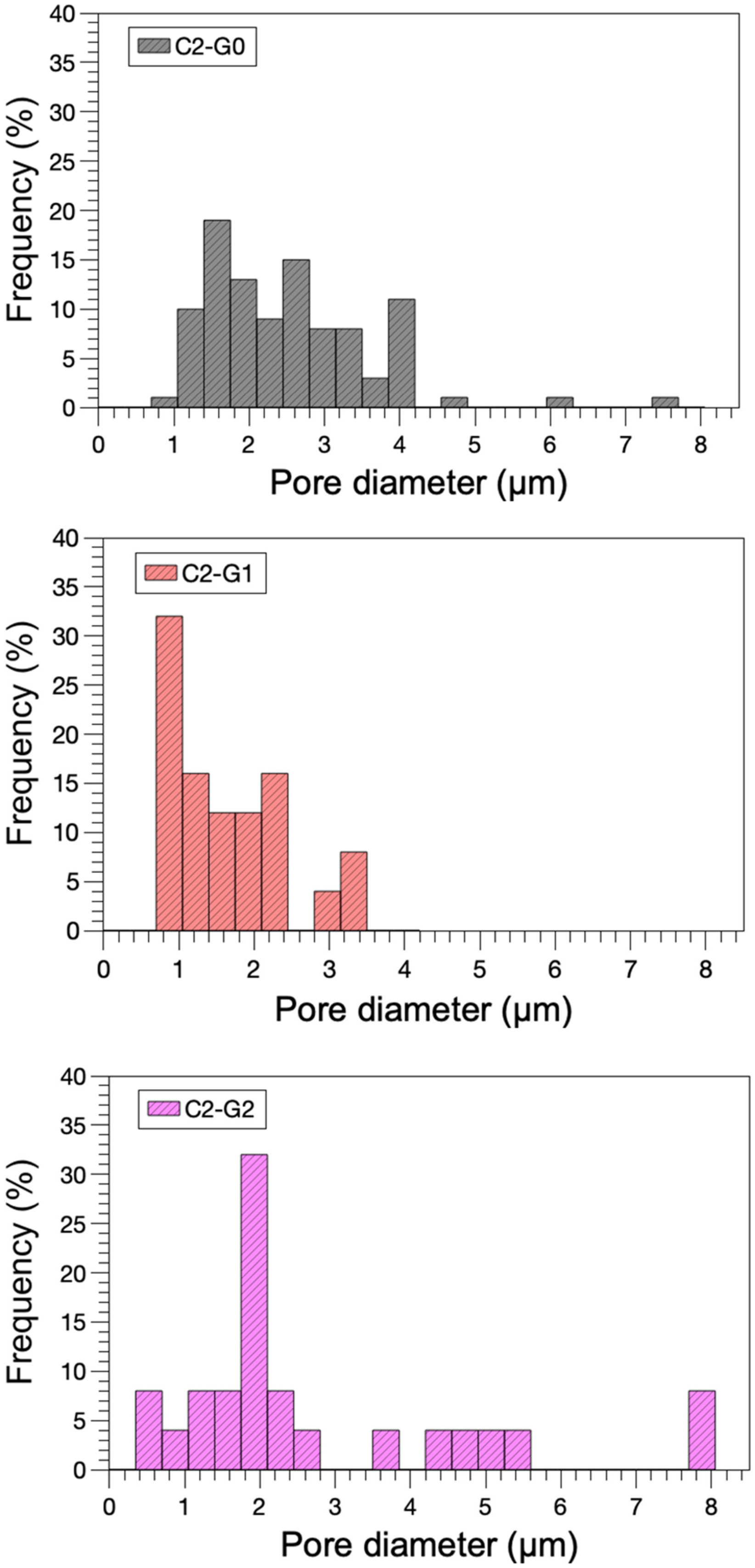

Figure S3. Pore diameter distribution of the membranes measured by Image analysis on the top surface.
